# Supplementary material for: Stacking Model‐Based Classifiers for Dealing With Multiple Sets of Noisy Labels
Source: Biom J. 2025 Mar 12;67(2):e70042. doi: 10.1002/bimj.70042 (PMC11898607; doi:10.1002/bimj.70042)
Supplement: Supplementary file 1 — Supporting Information [file BIMJ-67-e70042-s001.zip › density-based-ensemble-model-main/competitors_real_data/netlab-master/netlab-master/help/demolgd1.htm]

Netlab Reference Manual demolgd1


# demolgd1

## Purpose

Demonstrate simple MLP optimisation with on-line gradient descent

## Synopsis

```
demolgd1
```

## Description

The problem consists of one input variable `x` and one target variable
`t` with data generated by sampling `x` at equal intervals and then
generating target data by computing `sin(2*pi*x)` and adding Gaussian
noise. A 2-layer network with linear outputs is trained by minimizing a
sum-of-squares error function using on-line gradient descent.

## See Also

`demmlp1`, `olgd`

---

**Pages:**
Index


---

Copyright (c) Ian T Nabney (1996-9)
